# COVID-19 Disease and Vaccination: Knowledge, Fears, Perceptions and Feelings of Regret for Not Having Been Vaccinated among Hospitalized Greek Patients Suffering SARS-CoV-2 Infection

**DOI:** 10.3390/idr14040063

**Published:** 2022-08-08

**Authors:** Petros Ioannou, Sotiris Tzalis, Eirini Pasparaki, Despoina Spentzouri, Myrto Konidaki, Ioanna Papakitsou, Nikolaos Spernovasilis, Nikolaos Papanikolaou, George Samonis, Diamantis P. Kofteridis

**Affiliations:** 1 COVID-19 Department, University Hospital of Heraklion, 71110 Heraklion, Greece; 2 COVID-19 Department, Venizeleion General Hospital of Heraklion, 71409 Heraklion, Greece; 3 German Oncology Center, Limassol 4108, Cyprus; 4 School of Medicine, University of Crete, 71500 Heraklion, Greece

**Keywords:** COVID-19, vaccination, SARS-CoV-2, perception

## Abstract

Background: The development of vaccines against COVID-19 has greatly altered the natural course of this infection, reducing the disease’s severity and patients’ hospitalization. However, hesitancy against vaccination remains an obstacle in the attempt to achieve appropriate herd immunity that could reduce the spread of SARS-CoV-2. The aim of this study was to investigate the perceptions and attitudes of COVID-19 patients hospitalized during the fourth pandemic wave in two Greek hospitals and assess whether their experience had changed their intentions regarding vaccination against COVID-19. Methods: This is a cross-sectional, questionnaire-based survey, conducted from 31 August 2021 to 18 February 2022 in the COVID-19 departments of two tertiary care hospitals. The questionnaire included questions regarding the patients’ educational level, knowledge and beliefs regarding SARS-CoV-2, personal protection measures, beliefs regarding vaccination, vaccination status, reasons for not been vaccinated against SARS-CoV-2, feelings of regret for not been vaccinated, and willingness to be vaccinated in the future. All adult patients with COVID-19 were eligible, regardless of their vaccination status against SARS-CoV-2. Results: In total, 162 patients agreed and participated in the study, with 97% of them suffering severe COVID-19. Their median age was 56 years, and 59.9% (97 patients) were male. Among them, 43.8% had been vaccinated against COVID-19. When unvaccinated patients were asked the reasons for not being vaccinated, the most frequent responses were that they were waiting for more scientific data, due to uncertainty about long-term consequences of the vaccine, and their fear of thrombosis. When at discharge, unvaccinated hospitalized COVID-19 patients were asked whether they would get vaccinated if they could turn time back, and 64.7% of them replied positively. Conclusions: The study reveals several patients’ fears and misconceptions and suggests that there is room for implementing measures that could reduce knowledge gaps allowing for improvement of vaccination rates against COVID-19.

## 1. Introduction

Severe acute coronavirus 2 (SARS-CoV-2), the cause of coronavirus disease 19 (COVID-19), has so far infected more than 570,000,000 people, while more than 6,400,000 have died from this disease [1]. COVID-19 has caused multiple dramatic consequences in human health, leading to hospitalizations and medical complications, associated primarily with the respiratory tract, and significant mortality, while, on the other hand, it has caused significant consequences to the global economy [2]. The development of vaccines against SARS-CoV-2 has greatly altered the natural course of the disease and has led to a reduction of the disease’s severity and patients’ hospitalizations [3,4,5]. However, hesitancy for vaccination remains an obstacle in the attempt to achieve appropriate herd immunity aiming to reduce the spread of SARS-CoV-2 [6,7,8].

The use of social media has been on several occasions associated with public misinformation leading to detrimental effects such as denial of vaccination [9]. Some social media have fueled negative attitudes towards vaccination. These media were promoting ideological isolation of people who were selectively exposed to specific information of ambiguous quality, mainly provided by specific anti-vaccine groups [10,11]. In a study performed in Greece investigating behavior regarding seeking information about COVID-19, the majority of respondents were seeking information mainly by television, electronic press, and news websites, while the use of social media was limited [12]. However, even the mainstream media have sometimes led to the misinforming of the public, such as with an over-projection of adverse vaccine effects or by inviting scientists for commentaries whose scientific expertise was not related to COVID-19 [13]. Interestingly, a report assessing the presentation of COVID-19 and flu by Greek media, early during the COVID-19 pandemic, showed that up to 30% of the articles were misinterpreting the topic by providing misleading or deficient information, making it hard for the reader to detect false information [14]. Distribution of misleading information by Greek media is, unfortunately, not a new concept, as shown in a relatively recent study [15]. For example, by presenting conversations as news, instead of using data, opinions are preferred, often leading to the production of fake news [16]. This erroneous behavior may enhance misinformation regarding healthcare, leading to increasing hesitancy towards vaccination against COVID-19.

COVID-19 in unvaccinated patients can have a more severe course, often necessitating hospitalization and intensive care unit (ICU) support [17,18,19]. Moreover, COVID-19 disease among unvaccinated patients may lead to feelings of regret for not having been vaccinated earlier [20].

In Greece, a southern European, Mediterranean country with a population of about 10,400,000 people, as of 20 July 2022, 4,210,771 COVID-19 cases and 30,707 related deaths have been reported to the World Health Organization (WHO) [1]. Thus, until then, reported infections per 100,000 people were 39,284.84 and deaths per 100,000 people were 286.48. As of 25 June, a total of 21,161,653 vaccine doses against COVID-19 had been administered [1]. Until July 2022, 72% of the Greek population had been fully vaccinated against COVID-19, while 77% of the Greek population had received at least one vaccine dose [21].

The aim of this study was to record the perceptions and knowledge regarding SARS-CoV-2, as well as the fears and attitudes regarding vaccination for COVID-19 of patients hospitalized for this disease during the fourth pandemic wave in two Greek hospitals. AN additional aim was to assess whether hospitalization for COVID-19 could cause feelings of regret for not been vaccinated against and could change patients’ intentions to be vaccinated against this disease in the future.

## 2. Materials and Methods

This is a cross-sectional survey based on a non-validated questionnaire conducted from 31 August 2021 to 18 February 2022 in the COVID-19 departments in two tertiary care hospitals in Heraklion, Crete, Greece. All adult patients with COVID-19 were eligible to participate. All consecutive patients admitted to these departments were asked to participate, and thus sampling was non-probabilistic. Patients were considered to have severe COVID-19 according to the Infectious Diseases Society of America (IDSA) criteria [22].

A printed questionnaire was developed by a team of infectious disease specialists, fellows, and internists. It consisted of 20 items, including close-ended, multiple choice, and Likert scale questions. Its contents included questions regarding the patients’ educational level, knowledge and beliefs regarding SARS-CoV-2, personal protection measure use, beliefs regarding vaccination and their vaccination status, reasons for not been vaccinated against SARS-CoV-2, feelings of regret for not been vaccinated, and willingness to vaccinate in the future. The questionnaire is available in the Supplementary Material. Participation was voluntary, anonymous, and without compensation. The patients were invited to participate through direct contact by a study investigator. Informed consent was distributed concomitantly with the questionnaire.

Descriptive statistics were performed with GraphPad Prism 6.0 (GraphPad Software, Inc., San Diego, CA, USA). Qualitative data were presented as counts and percentages. Continuous variables (age) were initially assessed for normality with the D’Agostino and Pearson omnibus normality test and were then presented as means with standard deviation, as they were normally distributed. Quantitative variables were compared using Student’s t-test for normally distributed variables and the Mann–Whitney U-test for non-normally distributed variables. Statistical analysis of qualitative data was performed through contingency analysis with a chi-squared test. All tests were two-tailed, and *p*-values < 0.05 were considered to be significant.

## 3. Results

In total, 228 patients were asked to participate in the study, and finally, 162 (71.1%) agreed and participated. Among them, 69.8% (113 patients) were hospitalized in the University Hospital of Heraklion, and the rest were hospitalized in the Venizeleion General Hospital of Heraklion. Median age was 56 years (interquartile range: 47 to 71 years), and 59.9% (97) were male. Severe COVID-19 was diagnosed in 97% of enrolled patients.

**Figure 1:**
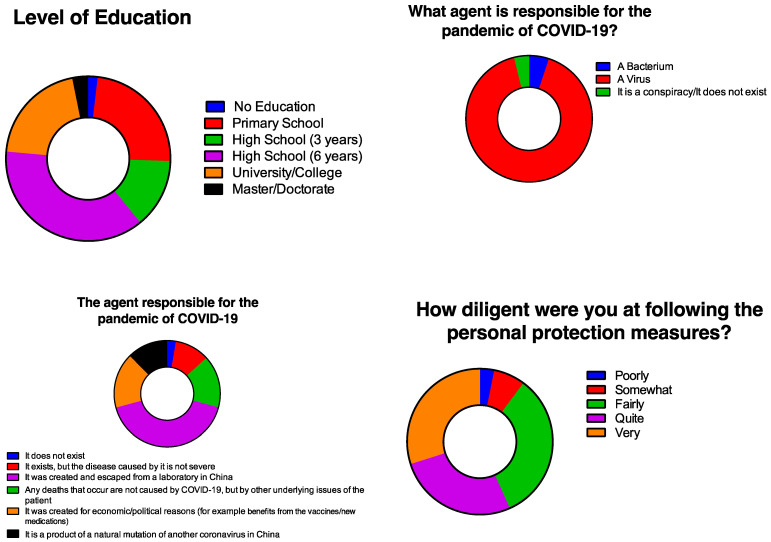
Perceptions and knowledge about COVID-19.

Figure 1 summarizes the educational level of patients and shows their perceptions and knowledge about COVID-19. In terms of education, 23.6% of them had completed only primary school, 37.3% had completed six additional years of high school, while 20.5% had a university degree. When asked about the agent responsible for COVID-19, the vast majority (91.1%) responded that it is a virus. Furthermore, regarding the origin of the virus, most patients (41.6%) responded that the causative agent was created in, and escaped from, a laboratory in China. When asked about whether they had been following personal protection measures, most of them replied that they were following them at least fairly. In particular, 33.1% were fairly diligent; 26.8% were quite diligent; and 29.9% were very diligent, following strict personal protection measures.

**Figure 2:**
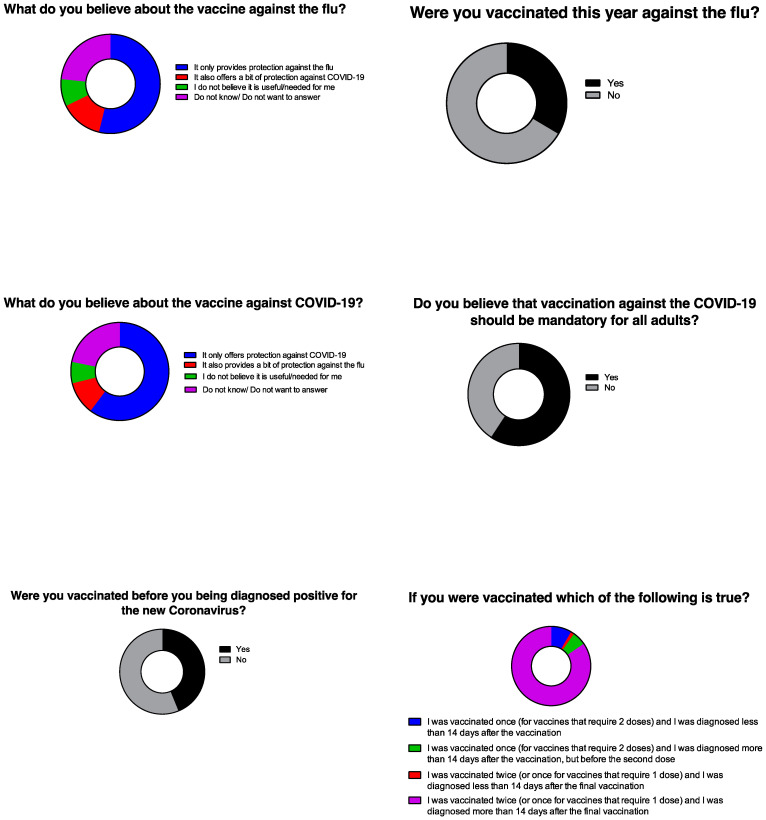
Perceptions on flu and COVID-19 vaccination.

Figure 2 summarizes the perceptions of hospitalized patients with COVID-19 about vaccination against flu and COVID-19. When asked regarding the flu vaccine, most of them (53.8%) responded that they believed it only provides protection against flu, and not against COVID-19, while 13.9% replied that they believed it also provides protection against COVID-19. When asked whether they had been vaccinated against the flu during late 2021 or early 2022, 66.5% replied that they had not. Furthermore, when asked regarding the COVID-19 vaccine, most of them (60.3%) responded they believed that it only provides protection against COVID-19, and not against flu, while 10.9% replied that it also provides protection against flu. When asked whether they had been vaccinated against COVID-19, 56.2% replied that they had not. When asked if vaccination against COVID-19 should be obligatory, 59.2% replied that it should. Most (68.7%) vaccinated patients had been vaccinated with the Pfizer BNT162b2 vaccine. Moreover, most patients (84.5%) had completed the vaccination at least two weeks before being diagnosed with COVID-19.

**Figure 3:**
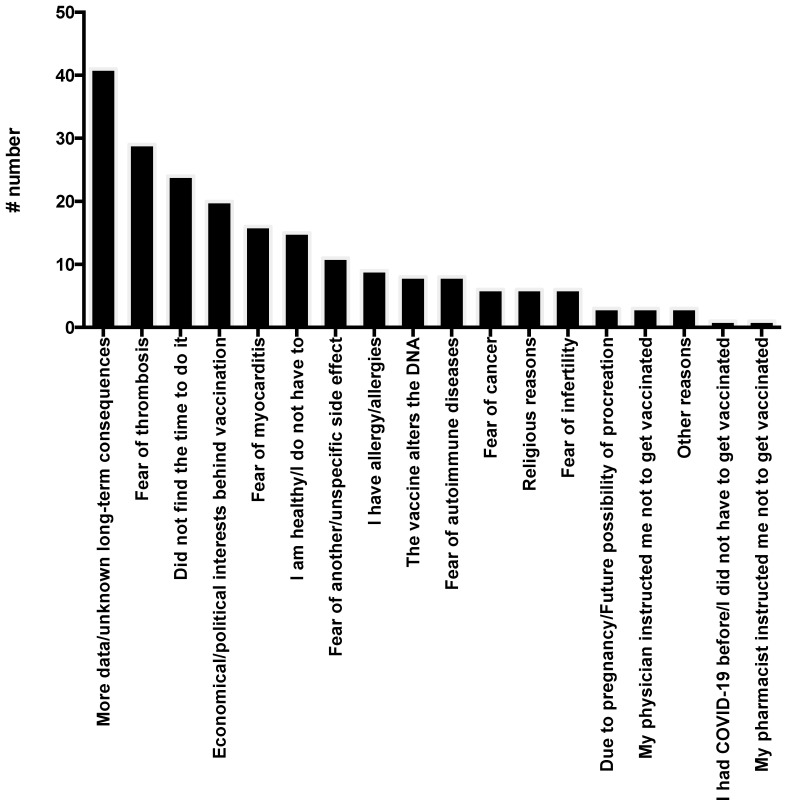
Reason for not having been vaccinated among unvaccinated patients hospitalized for COVID-19 (more than one answers possible).

Figure 3 shows the reasons why unvaccinated hospitalized patients with COVID-19 were not vaccinated. When they were asked the reasons for not being vaccinated (multiple answers were accepted), the most frequent responses included waiting for more data due to unknown long-term consequences of the vaccination (40.6% of them), fear of thrombosis (28.7%), being willing to get vaccinated but not having the time to do it (23.8%), and belief of economical/political interests behind the policy of mass vaccination (19.8%).

**Figure 4:**
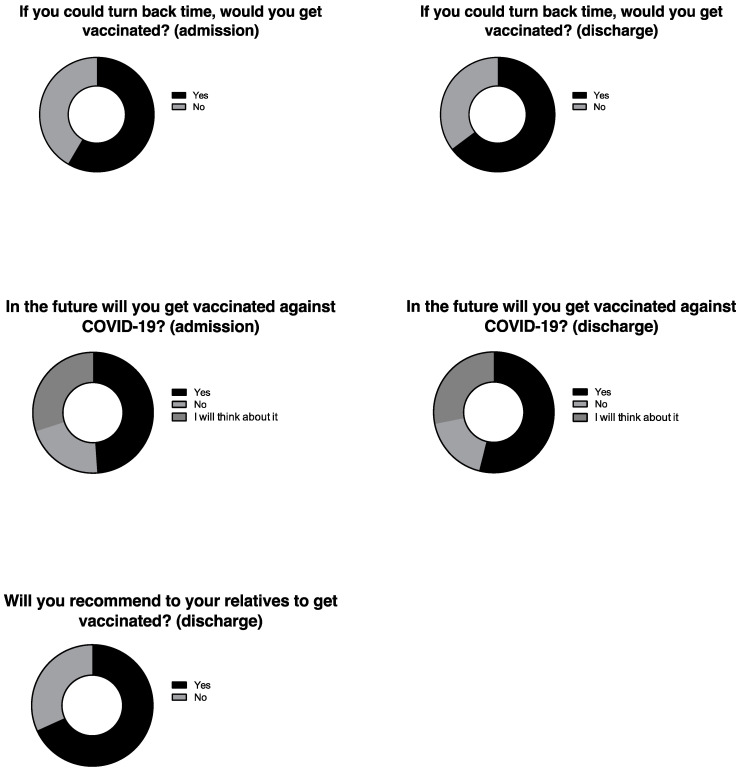
Willingness to be vaccinated among unvaccinated patients hospitalized for COVID-19.

When unvaccinated patients were asked whether they would get vaccinated if they could turn time back, 58.4% replied positively when asked upon admission, while 64.7% replied positively when asked on discharge. When unvaccinated patients were asked whether they would get vaccinated after discharge, 48.9% replied positively when asked upon admission, while 53.9% replied positively when asked on discharge. When unvaccinated patients were asked whether they would suggest vaccination against COVID-19 to their relatives, 68.2% replied positively (Figure 4).

Patients who were at least partially vaccinated did not differ in terms of gender from unvaccinated patients but they were older (median 65 years vs. 53 years, *p* < 0.0001). Unvaccinated patients were more likely to necessitate high-flow nasal oxygen (HFNO) therapy compared to patients who were at least partially vaccinated (42.2% vs. 14.3%, *p* = 0.0001) and had a non-statistically significant trend towards longer duration of stay in the hospital (median 9 days vs. 7 days, *p* = 0.0673).

## 4. Discussion

The present study examines the knowledge and perceptions regarding COVID-19 and vaccination against this infection among patients with COVID-19 hospitalized during the fourth pandemic wave in two tertiary hospitals in Heraklion, Crete, Greece. The study reveals the fears and misconceptions regarding the origin of SARS-CoV-2 and adverse effects of vaccination against COVID-19 and implies that there is a need and room for specific educational and other interventions that could reduce knowledge gaps and increase acceptance of vaccination in order to reduce COVID-19 adverse outcomes.

Public attitude can be crucial in the attempt to fight infectious diseases. Public knowledge and understanding of basic needs and scientific progresses regarding specific infections are important prerequisites, since acceptance of scientific advances and agreement to comply with voluntary measures, such as vaccination and isolation, can increase protection of public health measures leading to control of the disease [23]. To that direction, understanding the existing misconceptions and educational gaps can be of utmost importance leading to organization of interventions that can increase awareness and compliance to suggested practices [24].

In this study, most patients were at least fairly diligent with following personal protection measures and believed that the causative agent of COVID-19 was a virus. However, the majority of patients believed that the virus did not evolve naturally, but it was either created in a laboratory, or it was created on purpose for financial and social reasons, while many patients believed that many deaths were associated with underlying medical conditions, rather than being directly associated with the virus. It has already been observed that people often tend to underestimate the real threat of what appears to be a potentially life-threatening condition [25].

One of the basic aims of this study was to assess patients’ perceptions and attitudes towards vaccination against COVID-19. In order to have a comparison with COVID-19 vaccination, we also asked patients about their attitudes against flu vaccination. It is known from other studies that the COVID-19 pandemic has led to an increased acceptance of influenza vaccination [26,27,28,29]. Regarding the present patients’ attitudes, a significant proportion stated that the flu vaccine is not needed, or denied to answer. Furthermore, most patients had not been vaccinated against the flu, implying that there may be significant room for public information regarding flu vaccination. This is in line with studies showing that targets for vaccination against the flu are not met in patients’ populations with indication, or even in healthcare workers [29,30,31,32]. Similarly, a significant proportion stated that the vaccine against COVID-19 is not needed, or denied answering, and most of them had not been vaccinated against this infection. This hesitancy should be approached with attempts to understand the context of the relationship between misinformation, often from social media and unreliable sources, and associated emotional reactions [33,34,35]. To understand this, one must consider the social context of the population studied. In Greece, as shown in a recent study, the majority of the population was found to seek information regarding COVID-19 mainly by television, electronic press, and news websites, while the use of social media was limited [12]. Use of social media has been shown to be associated with the spread of misinformation, and this could affect society and healthcare, such as by increasing vaccine hesitancy [9].

Among the reasons for not being vaccinated, the most common causes stated by patients included the relatively limited data on long-term consequences and the fear of thrombosis. Indeed, fear of complications is a well-known factor associated with reduced willingness for vaccination against COVID-19, even among healthcare workers [36,37,38]. Thus, appropriate educational actions are needed to propagate the benefits of COVID-19 vaccination against possible risks and increase the confidence of public to healthcare providers and vaccination campaigns.

Interestingly, in this study, unvaccinated patients with COVID-19 were asked whether they would have been vaccinated if they could go back in time, and whether they will be vaccinated after being discharged, and most patients were positive. However, a proportion of them remained negative even after that. Similarly, most of these patients would also suggest to their relatives to be vaccinated as well. However, respondents’ self-reported future intentions do not necessarily mean they will indeed act as stated in the questionnaire.

This study has some limitations. First of all, it includes data from only two hospitals from the same region of Heraklion, Crete, Greece. Hence, generalization of the present results should be performed with caution. Furthermore, about 29% of the hospitalized patients with COVID-19 denied participation in this study, and this may imply that the study population may not be completely representative of the general population suffering SARS-CoV-2 infection. To that direction, it is important to note that this study included only hospitalized patients with COVID-19, and thus the perceptions and attitudes presented herein may differ from those of patients who were not hospitalized. Indeed, patients included in this study were suffering severe COVID-19, and their age may have been higher than that of non-hospitalized individuals with COVID-19. This could have an impact on the hospitalized patients’ attitudes and knowledge, since their educational level may not have been as high as that of younger patients suffering from non-severe disease. Furthermore, patients suffering severe COVID-19 may be more likely to regret not having been vaccinated compared to patients suffering non-severe COVID-19 [20]. Moreover, the questionnaire used in this survey has not been validated before. In addition, some questions did not provide an option to answer ‘I don’t know’, and this may have affected the results of the study. Finally, the study sample was relatively small, and thus further studies may be required for confirmation of the present results.

## 5. Conclusions

To conclude, this study shows that hospitalized patients with COVID-19 may have specific knowledge gaps and misconceptions regarding this infection, and vaccination against it that may be mostly associated with the origin of SARS-CoV-2 and the overrated possibility of adverse events from vaccination. Furthermore, a significant proportion of hospitalized COVID-19 patients, that had not been vaccinated before, seem to have regretted this decision and seem to be willing to be vaccinated after being discharged and recommend vaccination to their relatives as well.

## Data Availability

The data presented in this study are available on request from the corresponding author.

## Supplementary Materials

The following supporting information can be downloaded at https://www.mdpi.com/article/10.3390/idr14040063/s1, Questionnaire on vaccination against COVID-19.
