# Supplementary material for: COVID-19 Disease and Vaccination: Knowledge, Fears, Perceptions and Feelings of Regret for Not Having Been Vaccinated among Hospitalized Greek Patients Suffering SARS-CoV-2 Infection
Source: Infect Dis Rep. 2022 Aug 8;14(4):587–96. doi: 10.3390/idr14040063 (PMC9408697; doi:10.3390/idr14040063)
Supplement: Supplementary file 1 [file idr-14-00063-s001.zip › idr-1821753-supplementary.pdf]

Questionnaire on vaccination against COVID-19

1. Hospital:…………………………………..
2. Gender
    a. Male
    b. Female
3. Age:………………………………
4. Level of Education
    a. No Education
    b. Primary School
    c. High School (3 years)
    d. High School (6 years)
    e. University/College
    f. Master/Doctorate
5. What agent is responsible for the new Coronavirus Pandemic (COVID-19)?
    a. A Bacterium
    b. A Virus
    c. A Fungus
    d. It is a conspiracy/It does not exist
6. The agent responsible for the Coronavirus Pandemic COVID-19:
    a. It does not exist
    b. It exists, but the disease caused by it is not severe
    c. Any deaths that occur are not caused by COVID-19, but by other underlying issues of the patient
    d. It was created and escaped from a laboratory in China

    e. It was created for economic/political reasons (for example benefits from the vaccines/new medications)
    f. It is a product of a natural mutation of another coronavirus in China

7. How diligent were you at following the personal protection measures?
    a. Poorly
    b. Somewhat
    c. Fairly
    d. Quite
    e. Very
8. What do you believe about the vaccine against the flu?
    a. It only offers protection against the flu
    b. It also offers a bit of protection against COVID-19
    c. I do not believe it is useful/needed for me
    d. Do not know/ Do not want to answer
9. Were you vaccinated this year against the flu?
    a. Yes
    b. No
10. What do you believe about the vaccine against COVID-19;
    a. It only offers protection against COVID-19
    b. It also offers a bit of protection against the flu
    c. I do not believe it is useful/needed for me
    d. Do not know/ Do not want to answer
11. Do you believe that the vaccination against the COVID-19 should be mandatory for all the adults?
    a. Yes
    b. No

12. Were you vaccinated before you were diagnosed positive for the new Coronavirus?
    a. Yes
    b. No
13. If you were vaccinated, which vaccine were you vaccinated with?
    a. Pfizer
    b. Moderna
    c. Astra-Zeneca
    d. Johnson & Johnson
    e. Other
    f. Do not know
14. If you were vaccinated which of the following is true?
    a. I was vaccinated once (for vaccines that require 2 doses) and I was diagnosed less than 14 days after the vaccination
    b. I was vaccinated once (for vaccines that require 2 doses) and I was diagnosed more than 14 days after the vaccination, but before the second dose
    c. I was vaccinated twice (or once for vaccines that require 1 dose) and I was diagnosed less than 14 days after the final vaccination
    d. I was vaccinated twice (or once for vaccines that require 1 dose) and I was diagnosed more than 14 days after the final vaccination
15. If you were not vaccinated, which of the following were the reasons you were not? (More than one answers possible)
    a. I wanted to get vaccinated, but I did not find the time to do it
    b. I had already been sick with the COVID-19 before and I believed that I did not have to

c. I was waiting so we can have more data/ We do not know yet long-term consequences of the vaccination
d. I believe there are economic/political interests behind the mass vaccination
e. Due to pregnancy/Future possibility of procreation
f. I have allergy/allergies (Any)
g. Fear of thrombosis
h. Fear of myocarditis
i. Fear of cancer
j. Fear of autoimmune diseases
k. Fear of infertility
l. Fear of another/unspecific side effect
m. The vaccine alters the DNA
n. I believed that since I am healthy, I do not have to/ I do not belong to a high risk group for severe illness
o. My physician instructed me not to get vaccinated
p. My pharmacist instructed me not to get vaccinated
q. Religious reasons
r. Other reasons (describe)

16. If you could turn back time, would you get vaccinated? (admission)
    a. Yes
    b. No
17. If you could turn back time, would you get vaccinated? (discharge)
    a. Yes
    b. No
18. In the future will you get vaccinated against COVID-19; (admission)
    a. Yes

b. No

c. I will think about it

19. In the future will you get vaccinated against COVID-19? (discharge)

a. Yes

b. No

c. I will think about it

20. Will you recommend to your relatives to get vaccinated? (discharge)

a. Yes

b. No

21. Did the patient require admission in the Internal Care Unit (ICU)?

a. Yes

b. No

22. Did the patient require oxygen therapy with high-flow nasal cannula?

a. Yes

b. No

23. Days of Hospitalization………………..

Questions 21-23 to be answered by the physician
